# Prestige and gender role ideology: a study of young Tanzanian men

**DOI:** 10.1017/ehs.2025.4

**Published:** 2025-02-14

**Authors:** Alexander M. Ishungisa, Joseph A. Kilgallen, Elisha Mabula, Charlotte O. Brand, Mark Urassa, David W. Lawson

**Affiliations:** 1 National Institute for Medical Research, Mwanza, Tanzania; 2 Muhimbili University of Health and Allied Sciences, Dar es Salaam, Tanzania; 3 Department of Anthropology, University of California, Santa Barbara, CA, USA; 4 Human Behaviour and Cultural Evolution Group, College of Life & Environmental Sciences, University of Exeter, Penryn, UK

**Keywords:** cultural evolution, gender, social learning strategies, gendered conflict, global health

## Abstract

With the objective of informing theoretical accounts of social learning and gendered conflict, we explore the role of prestige in the formation of men’s beliefs about gender in a semi-rural but fast urbanizing community in north-western Tanzania. Using focus groups and participant observation, we contrast the extent to which young men view elders and men from the neighbouring city as prestigious, and the beliefs they ascribe to each category. Elders were viewed as prestigious because of their age and position as preservers and teachers of societal norms. Their prestige was culturally mandated, as evidenced by customs bestowing respect. In contrast, only subcategories of city men were deemed prestigious dependent on individual achievement. Prestige was difficult to distinguish from dominance, as both elders and city men can exert penalties on those with differing views. Elders were viewed as mostly, but not always, unsupportive of women’s empowerment, whereas city men were viewed as mostly, but not always, supportive of women’s empowerment. We conclude that urbanization shifts the distribution of prestige, exposing individuals to novel sources of social influence. However, future studies should be wary not to oversimplify elders as upholders of patriarchal beliefs and city men as universally supportive of women’s empowerment.

## Graphical abstract

**Figure:**
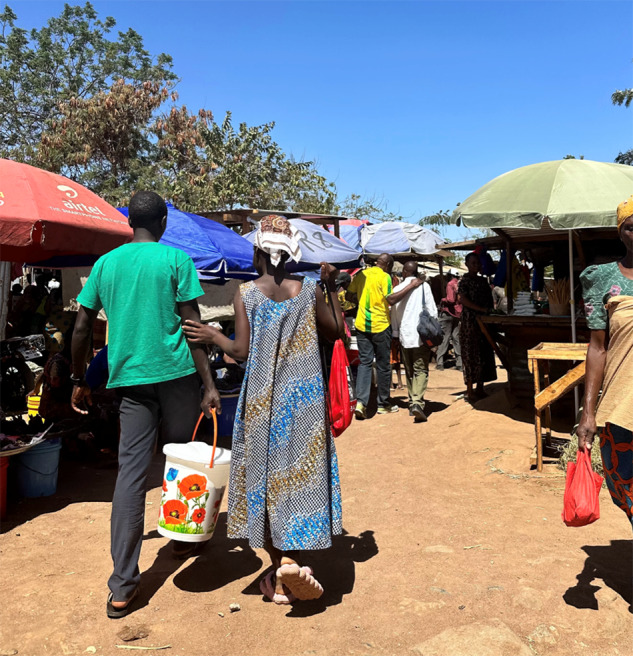

## Social media summary

Prestigious elders and urban men exert social influence on men’s beliefs about gender

## 1. Introduction

Evolutionary approaches to cultural evolution (Creanza, Kolodny & Feldman, 2017; Mesoudi, 2011) have been argued to have great potential to both account for human behavioural variation and inform global health initiatives (Alvergne, 2023; Gibson & Lawson, 2015). Key to this framework is the notion that evolution has bestowed us with discriminate social learning strategies or ‘transmission biases’ such that individuals selectively copy others based on the frequency or content of a behaviour, under certain ecological or individual circumstances, or based on the characteristics of the person or persons observed (Kendal et al., 2018). This latter form of social learning strategy constitutes model-based social learning and is exemplified by prestige bias (a tendency to preferentially copy individuals of high status). In this article, we explore the role of prestige bias in the social learning of gender role ideology, defined here as individually held attitudes about the appropriate roles, rights and responsibilities of each gender (Krosta, 2007). Within global health, gender role ideology is generally assumed to be a product of gender socialization, with individuals acquiring beliefs or ‘gender norms’ through a process of social learning and corresponding rewards and punishments for conformity and deviation, respectively (John, Stoebenau, Ritter, Edmeades & Balvin, 2017). There is also widespread recognition that global progress on tackling gender inequities is contingent on addressing prevailing patriarchal gender norms (Jayachandran, 2015). However, to date, few studies have explicitly applied the social learning strategies framework to gendered conflict, that is, evolutionary relevant conflicts of interest between women and men (Lawson, Alami & Somefun, 2023). The wider cultural evolution literature is also limited by a focus on formal mathematical modelling and experimental studies, which lack external validity (Mesoudi, 2011). Here, we aim to shed light on the role of prestige bias in the formation of young men’s beliefs about women and men via a novel field study in an urbanizing community in north-western Tanzania. We hypothesize that prestige is held by both community elders and relatively high-status community outsiders from the city, but that these groups will hold divergent beliefs about appropriate gender roles.

Prestigious individuals are generally defined as those who are ‘conferred deference’ (paid disproportionate respect/attention) due to being particularly skillful or knowledgeable in a given domain. In simple terms, a prestigious person would be one who is listened to, has influence on others, and whose opinions are heavily weighted because they enjoy credit, estimation or high standing in general opinion (Brand & Mesoudi, 2019). In the social learning process, individuals are hypothesized to copy behaviours of individuals who are highly respected and admired in their social group, that is, to copy ‘prestigious’ individuals (Brand, Mesoudi & Morgan, 2021; Henrich & Gil-White, 2001). The evaluation and determination of prestige, however, varies depending on the context and object towards which it is directed, for instance, by social position, occupation, age, education, wealth and so on (Brand et al., 2021; Burdett et al., 2016; Chellappoo, 2021; Mesoudi, 2011). In abstract theoretical terms, prestige can also be distinguished from dominance, in which dominant individuals acquire respect and attention, not through skill or knowledge but through fear and perceived or actual threats or intimidation (Henrich & Gil-White, 2019). Analytically, however, it can be difficult to separate these influences, at least outside of controlled experimental conditions (Jiménez & Mesoudi, 2021; Roberts, Palermo & Visser, 2019), because prestigious individuals may also engage in behaviours which exert social dominance, such as penalizing those who do not share the same values.

Tanzania, like many low- and middle-income countries, is urbanizing rapidly. This urbanization is also accompanied by shifting gender roles, such as increases in education for girls and women, and women’s labour market participation (Kilgallen et al., 2022). These changes place young people in urbanizing communities at a juncture; should they maintain prevailing gender norms or embrace relatively egalitarian perspectives on gender? Studying this question during adolescence and young adulthood is important because this life stage marks a critical period for identity formation (Bosma & Kunnen, 2001; Crocetti, Rubini, Luyckx & Meeus, 2008a; Crocetti, Rubini & Meeus, 2008b; Klimstra & van Doeselaar, 2017; Wohabie, 2019). Accordingly, we characterize our study as focused on young men as they are transitioning from late adolescence to adulthood, with all data collected from men aged between 18 and 30 years. Average age at first marriage in this context is in a man’s mid twenties (Marston et al., 2009), corresponding to relative independence and community recognition as a responsible adult. Furthermore, addressing the formation of gender role ideology among men is particularly important because men hold privilege under patriarchy, and typically hold more inequitable beliefs than women (Lawson et al., 2021a; Levtov, Barker, Contreras-Urbina, Heilman & Verma, 2014). By studying the dynamics of prestige in this context, we seek to both elucidate elements of the social learning process and generate knowledge capable of informing the design and evaluation of women’s empowerment intervention programmes. For example, a common intervention strategy is to enroll prestigious members of society to quicken the transmission of desirable beliefs or behaviours (Alvergne, 2023). To be successful, such an approach must first be grounded in a solid understanding of not only who holds prestige, but also community perceptions about the beliefs of prestigious people.

Our investigation takes a qualitative approach, with the aim of evaluating the following predictions. First, we predict that both community elders and outsiders from the city will be viewed as prestigious and, as such, influential. In rural environments, prestige is often assumed to lie in the hands of older high-status individuals (elders) (Gabriel, 2023; Wohabie, 2019) who play an important role in ‘vertical’ and ‘oblique’ social learning (from old to young) (Kendal et al., 2018). Throughout Africa, respect, particularly for elders, is considered a core value (Adeyemi & Adeyinka, 2002). Indigenous ideologies and socialization instill the value of respecting elders and others more than oneself. Some research conducted on this topic presents a generalized assumption that an individual’s level of respect increases with age, and therefore elders nearing death and ancestor-hood are more well respected than younger ones (Adeyemi & Adeyinka, 2002, 2003). One important reason why elders may be seen as relatively prestigious and influential is because they are holders and teachers of indigenous knowledge, teachers of land use, culture, identity, language, community history and spirituality, as well as the relationality of all living and non-living things (Adeyemi & Adeyinka, 2002; Reyes-Garcia et al., 2008). Generally, elders are viewed as repositories of societal norms, values and history to be passed on from one generation to another. Outsiders from the city may also be viewed as prestigious and influential. Urbanization increases exposure to prestigious outsiders in the form of relatively wealthy and more educated men in the city (Mutebi, 2022; Saint Onge, Hunter & Boardman, 2007), who may be important in ‘horizontal’ social learning (from those of the same generation) (Kendal et al., 2018). In this study, the term ‘city men’ refers to men of similar age who have received a relatively high level of education, secured good jobs and hold a good income as compared to other men in more rural communities. For city men, prestige is likely gained through education and occupation, as well as wealth accumulated (Arnett, 2002; Inglehart & Baker, 2000; Wohabie, 2019).

Second, we predict that elders will be viewed as relatively less supportive of women’s empowerment compared to young men. In other words, they will be viewed as having a relatively patriarchal gender role ideology. In protecting patriarchal norms, some studies have indicated that some elders in Ghana believe it better to die than support women, which they consider a dishonor (Adinkrah, 2012). Other studies in African settings have characterized opposition from elders upholding patriarchal traditions and attitudes as one of the barriers to achieving women’s empowerment (Adjei, 2015, see also Kilgallen et al., in press). However, not all research supports this perspective. Brooks and Blake (2021), for example, present a framework of ‘gendered fitness interests’ and argue that as men get older, they should care less about upholding patriarchal norms as they have already secured their own family and now are wary of potential negative effects of patriarchy on their less-privileged daughters. In a previous study of this population, we found no relationship between age and men’s support for women’s empowerment in a survey of men aged 20–45 years old (Lawson et al., 2021a). Cross-national studies of men’s support for gender equality have also reported inconsistent relationships with age. Overall, the relationship between age and men’s gender role ideology remains poorly understood.

Third, we predict that city men will be viewed as relatively supportive of women’s empowerment. In other words, they will be viewed as having relatively egalitarian beliefs about gender roles. This is because city men are generally more educated and exposed to external cultural norms as they interact with people from different ethnic and national backgrounds via urban life. Globalization, in this sense, may disrupt prevailing values and lead to the adoption of new beliefs (Berry, 2008; Wohabie, 2019). Supporting this conjecture, greater education and relatively high-status occupations, found more often in urban environments, have been associated with greater support for women’s empowerment and gender equality among African nations generally (Charles, 2020) and Tanzanian men specifically (Lawson et al., 2021a; Palermo, Chzhen, Balvin & Kajula, 2020). However, urbanization is a gradual process, and as it occurs, some people may change while others may avoid change to preserve their social status and protect their societal norms (Inglehart & Baker, 2000). Moreover, social learning hinges on (mis)perceptions about the beliefs of others, rather than the actual beliefs of others, which are obscured to some extent (Ishungisa et al., in press; Lawson et al., 2024). As such, it is critically important that we address how young men *perceive* others if we are to fully appreciate the role of prestige in the social learning process.

## 2. Methods

### 2.1. Study context

Data in this paper come from a semi-urban town in the Mwanza region located within the Magu Health and Demographic Surveillance Site (HDSS), which is managed by the National Institute of Medical Research (NIMR) (Urassa et al., 2024). Although other ethnic groups have moved to the area as result of urbanization and globalization, most of residents in the area belong to the Sukuma ethnic group (Wijsen & Tanner, 2016). Traditionally, the Sukuma were agropastoralists, and the practice continues today, even though both men and women are increasingly working in different livelihood activities; for instance, as skilled labourers, petty traders or in small businesses (Lawson et al., 2021a). Unlike in the recent past when they were busy with farm and pastoral work, young men currently spend much of their time in schools, business and labour work, striving to earn money which has become an important means to survive (Hedges, Sear, Todd, Urassa & Lawson, 2018). Although young men may primarily socialize with those of similar ages, as a semi-urban site, with a high population density, busy main road, market and many small businesses, they are frequently brought into contact with men of different ages, while also interacting regularly with elder men within their own families. Most young men, particularly if living or working in the town centre, also regularly interact with men from neighbouring, more urban areas, who come to the study community to work or trade.

Sukuma customs can be characterized as patriarchal (Wijsen & Tanner, 2016), with young men generally enjoying more support and favour than girls, and an expectation that adult men are the primary producers and owners of wealth, holding more power and decision-making authority than women. Boys are also expected to learn, hold and preserve community traditions and norms. Women and adolescent girls are traditionally expected to be engaged in household work such as cooking, taking care of children and other domestic chores. However, beliefs and behaviours regarding gender roles are changing, particularly in more urban areas, and are accompanied by a rise in both female education and labour market participation (Hedges et al., 2018; Kilgallen et al., in press). Attitudinal surveys carried out with men in the study community in 2019 revealed a diversity of apparent beliefs regarding gender roles (Lawson et al., 2021a). In general, men largely reported support for male authority in decision-making, and often condoned the use of intimate partner violence (IPV) to navigate spousal disputes (see also Kilgallen et al., in press), while also often declaring support for women’s labour market participation, participation in community meetings and girls’ education (Lawson et al., 2021a). For a broader discussion of changing gender norms in Tanzania see Badstue, Farnworth, Umantseva, Kamanzi and Roeven (2020), whereas Wijsen and Tanner (2016) provide an account of how Sukuma identity has been influenced by globalization.

For this study, data collection took place from June to October 2023 using focus group discussions (FGDs) and participant observation. Institutional ethical approval was granted by the University of California, Santa Barbara’s Office of Research (protocol number 7-23-0303), and the Tanzanian National Health Ethics Review Committee (NIMR/HQ/R.8a/Vol.IX/4359). Approval to conduct this study was also obtained at the community level following a presentation of the study objectives, requirements and expected outputs to community leadership.

### 2.2. Focus group discussions

A purposive sampling strategy was used to select FGD participants, targeting young men between 18 and 30 years old who had lived in the community for at least three months preceding the study. Recruitment of the participants was done by the first author, assisted by a field manager who was an employee of NIMR and familiar with the community. In each FGD, the number of participants ranged between 6 and 10 to gain effective participation (i.e. every participant in the group could have chance to share his perspective). Right after recruitment, participants were led through a consent process culminating in collection of signed consent. Participants were also given a hard copy of a study information sheet and, if they agreed to take part, were reimbursed for their travel to the FGD. During the discussion, which lasted between 60 and 90 minutes, study participants were provided with refreshments. Discussions were capped at 90 minutes to avoid participant fatigue and maintain data quality. All FGDs were conducted by the first author in Swahili, assisted by the third author. The FGDs were audio-recorded and later transcribed verbatim and translated into English for analysis and publication.

After introducing the topic at hand, discussions were directed to reveal perceptions about peer gender role ideology (the subject of a distinct manuscript: Ishungisa et al., in press), followed by questions asking participants to elaborate on perceived beliefs of elders and city men (the subject of the present paper). Discussions were kept on track by utilizing a discussion guide that focused discussion on two themes. The first theme addressed perceptions about what community elders/men from the city believe about changing roles of women and men. Example prompts in this theme included ‘What do they [elders/city men] think about men participating in household work, or caring for young children, is this something they think it is OK or good for men to do, or do they disapprove?’ and ‘What do they [elders/city men] think about women having control over their movements, for example, would they be happy to allow their [future] wives to leave the home to visit friends and family whenever they want? Or would they be unhappy about this?’ The second theme addressed how young men derived these beliefs about their elders and men from the city. Example prompts in this theme included ‘What actions of these men tell you they think this way, can you think of examples of things you have observed them doing that confirm your beliefs?’ and ‘Do you feel certain about the beliefs of these men? Or is it more of a suspicion? Why are you confident/not so confident about what they think?’ Discussions were approached with some degree of flexibility, so that the moderator could follow interesting observations as they arose rather than rely strictly on a script. As such not all dimensions of women’s empowerment were addressed in each FGD. FGDs continued until the first author felt saturation was reached. This led to a total of nine FGDs, with 72 participants in total.

Table 1 shows participant characteristics. FGDs were stratified by education and later religion. This stratification was done to allow variation of perspectives while also maintaining sufficient similarity in background within each group to foster effective discussion. The Tanzanian education system starts with primary education (7 years), then lower-level secondary (4 years), then high-level secondary (2 years), and thereafter an option to join a lower college for a certificate or diploma (2 years) or a university (with degrees taking between 3 and 5 years). In Table 1, ‘low education’ refers to only receiving primary education or some secondary, but not completing lower secondary (form 4); ‘form 4’ refers to completion of form 4 of secondary but not high-level secondary; ‘middle education’ refers to completion of high-level secondary or a lower college certificate or diploma; and finally, ‘higher education’ refers to those who had attended university. ‘Christian’ and ‘Muslim’ FGDs reflected purposive sampling of youth entrusted with religious leadership roles in their churches/mosques, such as religious youth leaders. Their education level varied from primary to college level.

**Table 1.** Characteristics of focus group participants

Focus group	Number of participants	Characteristics (all FGDs contained men aged 18−30, but were stratified by educational attainment or religion)
1	7	Mid-level
2	8	Lower
3	8	Form 4
4	8	College education
5	9	Lower
6	8	Form 4
7	8	College education
8	8	Christian
9	8	Muslim
Total	72	

### 2.3. Participant observation

Supporting data were gathered via participant observation (DeWalt & DeWalt, 2002), with the first author taking part in daily activities and events in the community that involved men aged 18–30. This was done to complement information gained through the FGDs and gain greater understanding of gender norms as lived, experienced and interpreted by young men. Participating in everyday activities and events further informed the first author about what young men perceive and believe about gender roles, and how prestige bias in the learning of gender role ideology is reflected in their everyday lives. Young men were joined in religious worship events, religious youth seminars, market gatherings, traditional ‘Bao’ games, bride price receiving events and the ‘amahane’ (a Sukuma male youth initiation event). At these activities the first author took brief notes that were later expanded into field notes. Observations are necessarily biased towards publicly observable behaviour, that is we cannot observe how young men interact with others in private.

### 2.4. Analysis

We took a directed content analysis approach (Hsieh & Shannon, 2005) to the analysis of FGD transcripts with our interest in prestige and social learning guiding data coding. The first author applied open coding to identify emerging themes using NVivo software, which we then later subsumed into broader thematic categories. For this paper, we draw on portions of the material that fell into categories we referred to as ‘prestige and social learning’, as well as elders and urban men’s beliefs. Although only the first author read all transcripts, co-authors who have previously investigated gender norms in the population discussed the coded material and took part in the selection of representative quotes. While we stratified focus groups by education and religion, this was primarily done to make participants more comfortable sharing their opinions with similar others. The present analysis is not concerned with differentiating men’s perspectives by these characteristics (which would likely require a larger number of FGDs), but rather the exploration of aggregate trends. Participant observation data also ensured confidence in the characterization of young men’s perspectives. In what follows, we summarize young men’s perspectives on peer gender role ideology and the confidence they ascribe to their perceptions. In our analysis we rely primarily on the FGD transcripts, but where appropriate mention subjective impressions based on participant observation.

## 3. Results

### 3.1. Elders are always prestigious, whereas for city men prestige is conditional on achievement

Focus group participants identified both elders and city men as having important degrees of respect and influence in the social hierarchies of their community. For elders, this prestige was viewed as a near universal trait, with a strong norm of young people being encouraged to show respect for older individuals, particularly senior men. This was reinforced by special language and styles of communication when addressing elders, and commonly observed during participant observation. For men from the city, however, participants noted a difference between an idealized city man with high education, occupying good position and office, and high income, and the reality of men living in urban settings in which life continues to be difficult. Not all city men were seen as different from men living in more rural or town environments as they too are oftentimes struggling to make a living. During participant observation, it was also observed that men coming from the city to interact with community members (for business, for example) were not clearly distinguishable in visible signs of status, such as clothing.

you know, elders are the most respected members of the society, they are not peers like us or children, you cannot address them the way you want or the way we are talking here. They need to be addressed with respect and with a precise tone and language. Without doing that they will interpret everything as disrespect. (P8, FGD6)

You will know the degree of respect given to elders by seeing how they are greeted. The word ‘shikamoo’ is always used to signify respect to a person with older age. Children and women will always kneel down or bend when greeting elders. (P3, FGD7)

Many men in the city are not very different from us. Yes, they have education, but may be of the same age like us, have same problems like us, they don’t have wealth or properties like us. To be honest, most of them are struggling with life like us. (P3, FGD9)

For the purposes of our study, we concentrated our discussions with participants on the idealized version of the city men (as those who are relatively highly educated and have a good job). For both elders and these successful city men, prestige and influence were attributed to three interconnected factors. One was the degree of knowledge they have compared to others. Elders, in particular, were considered to be the repository of traditional knowledge of the society as well as responsible for passing on this knowledge to younger men. Although elders were viewed as having in-depth knowledge of the norms and traditions of the society, (successful) city men, in contrast, were viewed as having knowledge due to their greater exposure to education and globalization.

… elders have lived long in the community, they are the ones who know every history, norms and traditions of the society. They are the ones teaching us everything about our society, including how to behave. We respect them, and need to behave well when interacting with them. (P5, FGD8)

Those learned people [city men], know many things, because they have attended more education than most of us. We need to respect them, and you need to prepare yourself very well if you want to talk to them, you cannot address them as we are talking here. (P1, FGD5)

Second, prestige was influenced by position occupied in perceived social hierarchies. This was frequently related to occupation, leadership roles and responsibilities individuals may be charged with in the society. Although successful city men were viewed as people who occupy formal jobs such as teachers, researchers, community leadership and other professional positions giving them the ability to influence others in the community, elders occupied both professional positions and community leadership. At their age, many elders become ‘balozi’, and gain more chances of being respected and exerting their influence on others through this role. Note in the Tanzanian local leadership system, for every group of 10 households, there is a local leader called ‘Balozi’. Balozi work together within a hamlet and village leaders in administrative matters in their areas. Balozi are typically at least 50 years old. Elders were also recognized as having a disciplining and conflict-resolution role in the community. Participants noted that external messaging about women’s empowerment, such as via development campaigns and programmes, had been delivered via interactions with both city men and elders.

The learned people [city men] have more influence than other groups because they do not only have education, they also take most of positions in offices. If you want service, you have to interact with them, and or visit their offices. Some are Sukuma and some are not, but they tell us what to do. You are talking about women empowerment, but there were some people who came here also to talk about these things in the past, and they all belong to that group of learned people [city men]. They have voices, and are respected. (P6, FGD1)

They [elders] are influential and respected. Even when the people in the government have their things or agenda to bring to us, they will use the elders, especially ‘balozi’, together with those from that group of learned people [city men]. (P2, FGD9)

Third, and linked to social position, is the resources that both elders and city men may have that identify them as holding privileged status in society. Participants referred to the amount of income, and properties like cattle, land and others that were possessed by the members in the two groups. Participants also believed that elders had lived longer in the community and have been able to accrue more resources as compared to other members of the society. City men on the other hand, providing they had been successful, gain resources from their salaries and job opportunities that were understood be more expansive outside of the community. Additional evidence of city men’s prestige was the idea that many women wished to form relationships with men from this group hoping for financial assistance.

Those city men are not very different from us … But their difference is that they are learned, and can get jobs that gives them money for the long period of their life. Because they are sure of the earning, they can invest in business or in other sectors. They will be respected because they have education, salary and other resources. (P4, FGD3)

People in that group [elders] own huge pieces of land and houses here, some of them also have cattle. We rent their houses, and sometimes farm on their land. We buy land from them. They are powerful and there is no way you can do without them. (P9, FGD5)

In Kisesa, most of women want to marry or form sexual relationships with city men because they are supportive and have more financial capacity than other men. Even if you become his [a city man’s] side (other) woman (‘mchepuko’), he is likely to finance you and your business. And if he doesn’t have capital, he is likely to give you courage and supportive ideas and strategies. (P6, FGD4)

### 3.2. Elders were mostly, but not always, viewed as relatively unsupportive of women

During discussions, young men generally identified elders in their community as unsupportive of women’s empowerment. Culturally, elders represent those in society that were raised when traditional norms and values were very strong, and currently this group stands as a repository of societal norms and traditions and are expected to preserve such norms amidst the rapid globalizing and urbanizing context where interaction with people of different cultures has increased. They are also expected to socialize the younger generation with these societal norms and traditions. Traditional life is viewed as having always been patriarchal, favouring men over women. Above all, elders were regarded as believing that supporting women might ruin their power and position in the society.

To be honest, no elder is supportive to women. They were raised and socialized when norms are stronger and they are here to protect these norms and traditions of the Sukuma society. Every elder knows that supporting women will ruin men’s power, respect, position, voice and privileges. Unlike other groups, elders do not support women. (P6, FGD1)

Elders are not supportive to women at all. They are the ones keeping the norms and traditions, and Sukuma norms don’t favour or support women. (P3, FGD9)

*Elders still hold selfish attitudes that mostly oppose women’s development and empowerment. They think that if women are empowered, they will take their positions and privileges in the society. That fear makes them [elders] remain selfish. But that is because they are less educated. (P7, FGD4)*

In general, elders were seen as less supportive to women in aspects such as allowing women to have their own income, education, leadership, deciding about having sex and children, divorce and marrying another wife. They were also less supportive of men’s participation in domestic chores. However, in some respects elders were deemed relatively supportive of women. It was alluded to clearly by study participants that unlike other groups of men in the society, elders were ready to allow their wives and female spouses to travel far away to visit friends and families. This behaviour was attributed to several factors. One was that elders had a long experience of living with their female spouses, allowing them to establish trust.

Those [elders] are different from other men …, … they are the ones that can allow their wives to travel far to visit their families and friends. Most of them are not jealous, have lived longer with their spouses, and they trust each other because they know each other’s behaviour. (P1, FGD4)

Second was the belief that men tend to age faster than women, and at their old age they surrender their working abilities and autonomy to women. Because of this belief, elders would offer women more support, such as in permitting them to travel far, not only for the purpose of visiting relatives, but to earn an income to support them and their families. Third, and connected to the second reason, was that at their old age and worsening health, elders lose their autonomy, power and control of their female spouses and hence women will be able to travel far even against the wish or permission of their male spouse.

… the situation [their weakening health] forces them to support women. You know men get older and die earlier than women. Men are unsupportive when they have energy, but when their age is gone, they lose energy, money, power and control. Women become breadwinners for the family and men are forced to be supportive. (P8, FGD5)

… at their old age, elders depend on the support from their wives. Most men know that the likelihood of dying earlier than their wives is high, because we have vivid examples here. Therefore, when they get old, they leave everything to their wives, women become powerful and have control of household affairs, income, and can travel wherever they want. A man has to respect that if he wants to live a peaceful and less stressful life. (P3, FGD6)

Some elders were also pictured as somewhat supportive of women’s empowerment more generally. Two reasons for this were that they become supportive either to grab opportunities such as employment, or they have married educated and affluent women and lost control to them, hence being supportive becomes a new coping strategy. In these cases, skepticism was raised about whether these men were genuinely supportive of women.

Some elders pretend to be supportive to women, but in reality, they are not. Those who have married young or affluent women, lose their control to these women. These women will do whatever they want even when they have no approval from their men. (P2, FGD3)

Some elders were also understood to have been exposed to education, globalization and interaction with other non-Sukuma ethnic groups, which was thought to influence their views. These elders are themselves supportive of women’s empowerment and influence other elders in the community to support women’s empowerment. This change is reflected through some elders increasingly supporting education for women.

I can say also the group of elders is divided, some are supportive and some are not. In this group there are educated people who have retired, or have exposure [to outside influences]. These [elders] are supportive to their women. But others are not supportive because of the traditions. (P7, FGD4)

In this sense, the introduction of the government and global policies that emphasize gender equality and education for all, including girls, was discussed as changing the attitudes of elders to be more supportive of women than in the past. Currently, many girls are enrolled in schools and supported by elders. However, some schoolgirls are still forced to quit school to get married where elders benefit from bride-price. There is also an increase of female employment in formal and informal sectors. For instance, as observed during participant observation, some women work as ‘balozi’ and are in charge of everyday administrative affairs of community members including elder men. Additionally, increase in support for women is influenced by the new government led by a female president. Through these global policies and the influence of the new government, some elders see many talents and abilities of women in all aspects of social, cultural, and political life and hence find the need to support women’s empowerment.

Modernization and urbanization are changing our norms and attitudes of people. It is true elders are not happy to support women in leadership, but the introduction of women’s rights and gender equality by the government is forcing them to accept. We see women in many positions now, even some balozi are women. (P6, FGD2)

Currently, we have a female president, and that has changed the mindset of many elders. They are listening and supporting women, and ready to give them leadership positions. (P5, FGD1)

Besides global policies and influence from the government, study participants pointed out that different projects coming into the community have also had a positive impact in changing elders’ attitudes towards women’s empowerment. A good example of these projects was the Tanzania Aids Monitoring Activities project (TAZAMA), which was run by the NIMR and has been running since the 1990s. Participants stated clearly that due to these interventions in the community, elders are gradually embracing supportive values towards women’s empowerment.

Different interventions coming in our community have been instrumental in changing elders’ views and attitudes. Some are now accepting women’s empowerment. We support what TAZAMA has been doing in our community, and we see many changed elders in the future. (P1, FGD8)

### 3.3. City men are mostly, but not always, viewed as relatively supportive of women’s empowerment

Participants generally pictured successful city men as more supportive of women’s empowerment as compared to other groups of men in the community. Here, the term city men referred to men who have received high levels of education and secured good jobs and earn a relatively good income as compared to other men in the society. They have also been exposed to city life, globalization and intercultural interactions. During discussions, participants pointed out that these men were relatively supportive of women’s empowerment compared to other men in their community.

men with education and position, are more supportive to their women than other groups in our community, because they have education and money. (P8, FGD2)

The rationale for this group being viewed as supportive to women was multifaceted. First, they have been exposed to education that transforms their traditional norms and attitudes to new ways of thinking, practices and perspectives towards women. Education was viewed as imparting to city men the required skills and values needed to support and cooperate with women.

Those with education, have exposure and skills on the importance of supporting women. They don’t even marry uneducated women, they want women who are educated, so they support each other. (P3, FGD7)

Second, city men knew various avenues through which women could be supported and empowered even when they don’t manage to secure formal jobs. This kind of support was visible in everyday interactions, where these men were seen supporting their jobless spouses by opening up a business for them to run and earn an income. Participants viewed this behaviour as a form of division of labour, ensuring that female spouses do not remain idle in the home but collaborate in the income generation for their family. Study participants also pointed out that many businesses run by women present in the study context were supported by these city men.

City men are highly supportive more than other groups of men. Even when their wives are not employed nor educated, they know how to use division of labour, they go to office, but start business for their women to make them busy and generate more income. (P7, FGD2)

Third, and linked to exposure to education, urbanization and globalization, study participants believed that city men are more supportive practically because they must have interacted with women of relatively high social status who have achieved greater things than their male peers. For instance, some of these women could be their university professors, mentors, researchers and so on. Also, because city men are exposed to multicultural interactions this was thought to reduce their egocentric (thinking of oneself and less of others) and ethnocentric (using one’s cultural standards to judge others) values and attitudes towards women and gender role ideology.

These men [city men] will have been not only transformed by education they get, the work they do and exposure to city life and media, but they will have also seen and interacted with many women with different talents and achievements than men themselves like female professors or researchers. All that will change their thinking and view of women, then they decide to be different by supporting women. (P3, FGD7)

Study participants were confident that city men gave priority to women to decide over many aspects of life such as decisions about household income, education, leadership, as well as divorce or separation. Unlike other groups of men, city men participated in doing domestic chores like cooking, washing and so on, even though they still did not want such acts to become public knowledge to protect their image and status.

Learned people can do domestic work more than other men, because they know the ideas of equality. Even when they don’t tell that in public to avoid being interpreted differently, they still help their wives with domestic work. They are supportive in many angles of life. They listen and give chance to their wives to decide over having sex, children, income, and many other things. (P1, FGD6)

As noted above, city men were seen as different to locals only if they had been successful in acquiring opportunities outside of the community. Participants were cognizant that urban residence did not always come with such success, rendering many city men quite similar to those living in relatively rural areas. As such, some city men were viewed as similar in their support for women’s empowerment. Participants also noted that influences from traditional cultural norms remain strong even for city men, leading them to resist change, and retain fear of losing control, respect, position and social privilege. Participants perceived cultural change as a gradual and non-linear process, such that norm change may lag behind rapid urbanization.

I can say that it is fifty-fifty in this group. There are those supportive to women but there are those who are not, we see them here. Some are transformed by education, exposure and globalization, they learn from other men in the globe, but others remain conservative, they keep the norms, and they are not supportive to women even their wives. (P4, FGD5)

Educated men do exist here …, but when it comes to supporting, they appear to be the same as other groups, they are less supportive, all men here hold the same beliefs. (P5, FGD8)

## 4. Discussion

Our first prediction was that both elders and men from the city would be viewed as prestigious by young men growing up in this fast-urbanizing semi-rural community. Our findings are consistent with this prediction, but also reveal distinctions between these two groups. Most importantly, although for city men prestige was considered dependent on men’s achievements, respect for elders was culturally mandated. For elders, prestige is not conditional on individual characteristics, but instead more about belonging to the respected social category of elder. This is illustrated by the use of special language and customs for talking with elders (e.g. ‘shikamoo’), and universal understanding that younger individuals must address elders in particular with a respectful tone and language so as not to be deemed disrespectful. These findings align with those from a study in Cameroon (Tchindjang, Bopda & Ngamgne, 2008), which also indicated that language plays an important role in prestige and identity formation among different social groups. The same study indicated that language shows how societal customs of specific groups are expressed. In our study, participants showed that some elements of elder’s status seem more akin to dominance than prestige, in the sense that there appears to be little choice in the deference given to elders. Elders are also involved in decisions and punishments as a way of enforcing norms and rules, suggesting that they may be viewed as not only prestigious but also dominant (Cheng, 2020). However, participants also highlighted factors such as knowledge and wealth, suggesting that prestige is operating in this group.

Our findings contrast with those of Reyes-Garcia et al. (2008), who found that age was not associated with prestige among sampled Tsimane communities in rural Bolivia. They speculate that this finding is not characteristic of ancestral patterns, but rather a product of recent changes. First, increasing contact with outsiders has introduced technological innovations to the Tsimane, leading the body of knowledge held by elders to depreciate in relative value. Second, declines in adult and old age mortality have increased the number of elders in the community, such that the accumulated knowledge of any individual elder is rendered less valuable. Such explanations are plausible, and our informal observations also suggest that the notion of mandatory respect for elders may be dissolving in Tanzanian cities. However, given that our study population is also characterized by similar transitions accompanying urbanization, further research is clearly required to fully understand what factors cause the prestige (and dominance) of elders to vary across time and space. From a methodological standpoint, it would be particularly illuminating to capture shifting perceptions of elders with longitudinal data as communities change with urbanization and outside influence. We also advocate for the value of mixed methods; as highlighted in our introduction, cultural evolution research remains dominated by abstract formal modelling and quantitative hypothesis testing. Qualitative research can play both a complementary and essential role in assuring assumptions about the dynamics of social learning are grounded in a solid understanding of ethnographic context.

Men from the city were also seen as prestigious, but this perception was much more contingent on them being financially successful and highly educated. In this study, a city man was pictured as a highly successful person, well-educated, occupying good and rewarding positions, and financially well off, making them very prestigious. Although this kind of man was well understood by study participants, who acknowledged that there were such men in the study context, we noticed that many city men lacked some of the above-mentioned features and hence were regarded as less prestigious compared to other non-city men in the community. Participants noted a shared understanding that urban life is challenging and thus not all urban men will be prestigious as they too are struggling to make a living just like men living in more rural settings. In this sense, respect for city men is conditional on individual characteristics rather than culturally mandated, as is the case for elders. For instance, although education was viewed as a means through which young men in cities gained prestige, study participants revealed that in a challenging urban life, some educated men were still struggling to get a job, earn an income and make a living, something that did not make them different from rural men who are not as exposed to education. When asked to elaborate as to how city men who are unsuccessful protect their respect and manhood, participants mentioned that these men may engage in lower-status work done by rural men out of necessity, such as quarrying, farming or motorcycle taxi services commonly known as ‘bodaboda’. The overall implication is that the concept of city men and prestige are not necessarily interlinked but instead conditional and context dependent.

Our second prediction was that young men would view elders as relatively less supportive of women’s empowerment. Consistent with our prediction, elders were *mostly* viewed as relatively less supportive of women’s empowerment as they protect and uphold patriarchal values and attitudes, are responsible for transmitting these values to the young generation, and have less exposure to external scripts about women’s empowerment via influences like education. However, there are important nuances here; not all elders were viewed as less supportive of women’s empowerment. Our analysis helps to identify several reasons why men may sometimes become *more* supportive of women’s empowerment as they age. First, trust between spouses is regarded as increasing over time, such that older men may feel less need to control their wives’ behaviour, as exemplified by a wife having the ability to travel away from the home. Second, at their age, elders have secured their families and are wary of potential negative effects of patriarchal norms for their less-privileged daughters. This pattern has been predicted from a ‘gendered fitness interests’ perspective, because as men age their inclusive fitness interests become increasingly distributed across both male and female kin (Brooks & Blake, 2021). Third, elder men may become increasingly dependent on their wives with age as they lose the ability to earn for themselves and families, and thus give that role to women or their female partners. This is also accompanied by the fact that men in this predominantly Sukuma community tend to marry younger women, thus wives are perceived as likely to remain strong when their husbands get old (Lawson, Schaffnit, Hassan & Urassa, 2021b). Under these circumstances, elders may afford greater control over the family to women out of necessity. This finding echoes work by Morgan and Brazda (2013) that indicates that at advanced age, elders in the United States lose control over tasks and decisions, which is shifted on to their caregiving kin or others. Our work helps to understand this rationale and reality in the Tanzanian context.

Our final prediction was that men from the city would be viewed as relatively more supportive of women’s empowerment. Consistent with this prediction, city men were *mostly* deemed relatively more supportive of women’s empowerment. This perception was associated with their exposure to education and globalization as well as their interaction with people from a multicultural background and diverse professions (see also Ishungisa et al., in press). Previous studies, including in this study population (Lawson et al., 2021a), have also confirmed that relatively well-educated men tend to declare greater support for women’s empowerment in quantitative attitudinal surveys, suggesting that such associations are somewhat generalizable (Charles, 2020; Levtov et al., 2014; Scott et al., 2014; Uthman, Lawoko & Moradi, 2009). However, here we also recognize differences between successful city men and the average struggling city man. In this urbanizing community, participants note that successful city men are able to demonstrate their support to women through practical actions, such as starting a small business for women, whereas struggling city men may support women if given the opportunity but could not demonstrate this support in practice as they lack the necessary capital. Participants acknowledge diversity among city men, noting that both those successful and those struggling may still cling to traditional patriarchal norms.

Although our study does not directly assess social learning (i.e. the extent to which young men’s beliefs and actions are influenced by elders or city men), it does provide valuable insights into the mechanisms of gaining and potentially losing prestige over time, as the social environment changes. In this vein, we suggest the following topics as fruitful areas for further research.

First, one revealing aspect of this study is that elders are not only seen as prestigious but potentially dominant too, as their respect is seen as mandatory, and they are involved in punishment and reward in the community. If this is the case, it could mean their status is somewhat protected, as even if prestige is lost over time (due to lacking education/exposure to the latest information) they may keep their influence via dominance, that is, by using fear or threat of punishment (Cheng, 2020; Scott et al., 2014). In hindsight, our investigation would have benefitted from structuring our discussions and observations to better distinguish the extent to which both elders and city men attain social status and influence from prestige (acquired skill and knowledge) rather than from dominance (via coercion and intimidation). As noted in our Introduction, it is difficult to separate these pathways in observational studies because they typically covary, perhaps particularly in relatively gerontocratic and patriarchal societies were power is unevenly distributed. On the basis of what data we do have, it seems that elder men in particular are viewed by younger individuals as both powerful and capable of exerting punishments on those who counter traditional values, with several of our selected quotes implying social sanctions if elders are not paid sufficient respect. However, privileged urban men also have capacity to exert influence via behaviours such as restricting employment opportunities for young men. It would be instructive to dedicate research into comparing how such sanctions are enacted on young men, which could include documenting the experiences of men whose values and behaviours oppose those of elders or high-status urban men.

Second, it would also be valuable to further explore the domain-specificity of prestige bias (Barnd & Mesoudi 2019, Brand et al., 2021; Morgan, W. & Griffiths, 2022). Elders’ prestige appears to be associated with upholding traditional social norms and values, thus community members may rely on them for behaviours relating to these norms specifically, but not other areas of life. In contrast, the prestige of men from the city appears to be associated with education and wealth specifically, so it could be predicted that community members look to city men for guidance on navigating novel dilemmas presented by urban life. It would be interesting to explore how such dynamics may play out with respect to women’s empowerment, given the relative novelty of phenomena like women’s labour market participation and involvement in senior leadership.

Finally, a third area of interest is the contrast between explicit advice giving and copying. Recent work highlights distinctions between adhering to explicit advice rather merely copying observed behaviour (Hertz, Bell & Raihani, 2021). Giving advice, for example, is associated with potential reputational costs, thus long-term advice-givers are associated with a high level of trust. This notion fits well with the position of elders in this study as they are seen as passing down advice to the next generation, which may instill high levels of trust in them in general. It would be instructive to study how this role of elders is shifting with urbanization.

Our study has limitations. Although data were collected by a Tanzanian research team and care was taken to make discussions comfortable places for sharing all perspectives, all team members are still outsiders to the community. Consequently, our discussions may have been influenced by some degree of social desirability bias. Potentially, young men may have felt motivated to portray others in their community favourably, downplaying low support for women’s empowerment across all groups. More generally, our data can only tell us how prestige is conceptualized and leave open questions about how both elders and city men actually interact with young men in their everyday interactions. Participant observation confirmed that interactions with elders and city men are commonplace, but it would be instructive to quantify the frequency and form of interactions. Future work may find deeper engagement with observational methods or individual in-depth interviews as well as field experiments useful. Finally, as with all qualitative research, this study did not access information about actual frequencies of views and beliefs of prestigious men. Here, we emphasize that perceptions about the beliefs of others may be just as influential, or even more influential, than what others actually believe in social learning. For example, in recent work we have documented widespread overestimation of peer support for inequitable gender norms, which may be acting to stifle transitions away from patriarchal beliefs in this context and others (Ishungisa et al., in press; Lawson et al., 2024).

## 5. Conclusion

Urbanization fundamentally shifts the dynamics of social prestige. In rural communities, prestige may be held among elders, but urbanization brings in novel role models via high status ‘city men’. This study supports this assumption, but also observes that forms of prestige are not symmetrical – for elders, prestige is relatively unconditional and culturally mandated, whereas for city men prestige is dependent on individual status and achievement. Our findings also suggest that we should be careful not to assume that elders are always less supportive and city men always more supportive of women’s empowerment. As noted by our participants, both elder and well-educated men from the city are often recruited by external agencies wishing to counter patriarchal gender norms. Policies, interventions and efforts targeting women’s empowerment can only be strengthened by first understanding variation in both the actual and perceived beliefs of these potentially influential groups. We also encourage scholars of cultural evolution to consider gender role ideology as a relatively neglected phenomenon, but with high potential to demonstrate the applied value of the social learning strategy framework.

## Research transparency and reproducibility

Data supporting our findings are directly reported in the manuscript (i.e. anonymized quotations from focus group transcripts). Full transcripts are not suitable for being made publicly available due to the sensitive nature of the content and the possibility of participant identification.
